# A deep hybrid CNN-BiLSTM-BiGRU architecture with explainability for mild cognitive impairment detection using EEG

**DOI:** 10.1186/s40708-026-00302-4

**Published:** 2026-05-11

**Authors:** Aishik Tokdar, Lakshya Agarwal, Shataghnee Chatterjee, Sukriti

**Affiliations:** School of Electronics Engineering, Vellore Institute of Technology, Chennai, India

**Keywords:** Mild cognitive impairment (MCI), Electroencephalography (EEG), Deep learning, Squeeze-and-excitation, Explainable artificial intelligence (XAI).

## Abstract

Accurate detection of Mild Cognitive Impairment (MCI) is critical for timely intervention and for slowing progression to Alzheimer’s disease. Electroencephalography (EEG) offers a non-invasive and cost-effective measure of brain activity; however, its complex, non-linear dynamics limit conventional analysis. We propose a CNN-Res-SE-BiLSTM-BiGRU framework for the automated detection of MCI directly from raw EEG. Convolutional and residual blocks capture local temporal structure, bidirectional recurrent layers model long-range dependencies, and Squeeze-and-Excitation (SE) modules provide channel-wise attention. Predicted probabilities are calibrated using temperature scaling, and operating thresholds are selected on the validation set using Youden’s J statistic. The model is evaluated using five-fold cross-validation under both subject-dependent and strict subject-independent protocols on a primary resting-state dataset, with additional subject-independent validation on an odor EEG dataset. Under subject-independent evaluation on the odor dataset, the proposed model achieved an accuracy of 0.956 ± 0.051, with ROC-AUC of 0.971 ± 0.051 and PR-AUC of 0.934 ± 0.132. UMAP-based visualization and explainable AI analyses (SHAP and LIME) provide interpretable insight into the learned spatiotemporal patterns and sample-specific decisions. These results demonstrate robust, interpretable EEG-based MCI detection with potential clinical utility.

## Introduction

Mild Cognitive Impairment (MCI) is increasingly recognized as a transitional clinical state between normal age-related cognitive decline and the onset of neurodegenerative disorders, particularly Alzheimer’s disease (AD) [1, 2]. It refers to early-stage cognitive impairment, often marked by isolated memory deficits or other subtle disturbances in domains such as executive function or language, that exceed the expected effects of normal aging but do not yet interfere significantly with daily functioning. MCI is considered a prodromal stage of AD, with a community prevalence estimated at approximately 21% in individuals over the age of 65. The global burden of cognitive disorders is substantial and growing. Currently, more than 46 million people worldwide are living with dementia, and this number is projected to rise to 131.5 million by 2050 [3]. As global life expectancy increases, the prevalence of MCI and its potential progression to AD poses a major public health challenge, placing a significant burden on individuals, caregivers, and healthcare systems. While epidemiological studies estimate that 10–20% of adults aged 65 and older show signs of MCI, only a fraction receive a timely and accurate diagnosis due to the subtlety of early symptoms. Early detection of MCI is crucial, as it enables preventive strategies to slow cognitive decline. It also enables individuals and their families to make informed decisions regarding lifestyle modifications, future care planning, and participation in therapeutic interventions and clinical trials.

To diagnose MCI, a variety of diagnostic tools are currently employed in clinical settings, including the Mini-Mental State Examination (MMSE), Computed Tomography (CT), Magnetic Resonance Imaging (MRI), Positron Emission Tomography (PET), blood tests, cerebrospinal fluid (CSF) analysis, and Electroencephalography (EEG) [1]. Neuroimaging techniques, such as PET, MRI, and CT, while providing valuable insights into both structural and functional aspects, are often resource-intensive and costly. MMSE, although popular, is a subjective, manually administered assessment that is prone to variability across individuals and examiners. EEG has emerged as a promising tool for MCI diagnosis due to its non-invasive nature, cost-effectiveness, and widespread availability. EEG also captures the brain’s electrical activity in real-time, with a focus on cortical oscillations that are crucial to understanding brain function. These temporal patterns provide vital information for detecting early-stage cognitive decline, thus making EEG a suitable candidate for the diagnosis of MCI [4]. Due to its practical advantages and diagnostic potential, this study utilizes EEG as a core modality for the early detection of MCI.

The processing of EEG signals has significant challenges. The recordings are susceptible to artifacts caused by ocular movements, muscular activity, and environmental noise. Additionally, EEG signals are intrinsically high-dimensional, exhibit non-stationarity over time, and require advanced analytical techniques for effective interpretation. Several previous studies have predominantly relied on traditional machine learning techniques, combined with handcrafted feature extraction, to differentiate between healthy controls (HC) and MCI patients. For example, Dauwels et al. [5] utilized Granger causality and stochastic event synchrony as features, employing linear and quadratic discriminant analysis (LDA/QDA) classifiers. Hadiyoso et al. [6] applied power spectral features and classified them using the K-nearest neighbor (KNN) algorithm. Kashefpoor et al. [7] employed spectral EEG features with a neuro-fuzzy algorithm combined with KNN classification. In their follow-up study, Kashefpoor et al. [8] introduced a supervised dictionary learning approach called correlation-based Label Consistent K-SVD (CLC-KSVD) for EEG signal analysis. Lee et al. [9] extracted a wide range of features, including power spectral density, entropy measures, asymmetry indices, and nonlinear dynamics, using a support vector machine (SVM) for classification. Hsiao et al. [10] proposed Kernel Eigen relative power (KERP) features and used Fisher’s method for feature selection, followed by SVM with leave-one-subject-out cross-validation. Yin et al. [11] applied the stationary wavelet transform (SWT) for denoising and extracted features through spectral and temporal analysis, then classified them with SVM. Siuly et al. [1] extracted features using auto-regressive modeling and permutation entropy, classifying them with an extreme learning machine (ELM). Finally, Movahed et al. [12] combined spectral, functional connectivity, and nonlinear features, and used a linear SVM for classification. However, the dependency on handcrafted features limits the adaptability and scalability of these models, because such features may fail to capture the complex, nonlinear nature of EEG signals completely. This might reduce their generalizability across different datasets and clinical settings.

In response to these limitations, deep learning models have been increasingly adopted to automatically learn feature representations from raw EEG signals. Convolutional Neural Networks (CNNs) are particularly effective for extracting spatial features. Ieracitano et al. [13] transformed EEG signals into power spectral density (PSD) images for classification, achieving 89.8% accuracy in tasks involving Alzheimer’s Disease (AD), Mild Cognitive Impairment (MCI), and Healthy Controls (HC). In a follow-up study [14], they introduced EEG-CNN, which processed pre-processed EEG signals directly, bypassing PSD conversion, and obtained 85.34 ± 1.86% accuracy for AD vs. HC classification. Kim et al. [15] developed CEEDNet, an end-to-end deep neural network for EEG classification, reporting ROC-AUC scores of 0.90 and 0.86 on dementia-related datasets. Given the sequential nature of EEG data, recurrent neural network (RNN) variants such as Long Short-Term Memory (LSTM) and Gated Recurrent Unit (GRU) have been employed to capture temporal dependencies. Alvi et al. [16] evaluated 20 LSTM models on a public MCI dataset, achieving up to 96.41% accuracy, while their GRU-based approach [17] reached 95.51%. Geng et al. [18] applied a Bidirectional GRU (BiGRU) to both sleep and awake EEG, obtaining a maximum accuracy of 93.46%. Rezaee and Zhu [19] enhanced CascadeNet with the discrete wavelet transform (DWT) and windowing for augmentation, reporting accuracies of 98.84% and 97.78% on two separate datasets. Hybrid CNN–RNN architectures, which jointly capture spatial and temporal patterns, have gained significant attention. Zhou et al. [20] introduced the Spatio-Temporal Convolutional GRU (STCGRU) network, which integrates three CNN modules with a BiGRU for raw EEG processing, achieving 99.95% accuracy through 10-fold cross-validation. Nirmala and Latha [21] proposed a Dual Attention Assisted Compact Convolutional Network with Stacked BiLSTM (DCCN-SBiL) for classifying EEG signals into AD, MCI, and HC. Their framework incorporates sequential Savitzky–Golay filtering for noise reduction, improved Tunable-Q Wavelet Transform (ITQWT) for feature extraction, Coati Stochastic Optimization (CSO) for channel selection, and a dual-attention CNN–BiLSTM classifier optimized via Gazelle Optimization Algorithm (GOA). The model achieved 97.25% accuracy for EEG-based MCI detection.

Recent advancements in MCI detection demonstrate a clear transition from traditional machine learning methods based on handcrafted features to automated, end-to-end deep learning approaches. Although models such as CNNs, RNNs, and their hybrid counterparts have achieved commendable accuracy, several limitations persist. Chief among these is the high computational complexity of hybrid architectures [19–21], which may hinder their integration into real-time clinical workflows. Despite these advances, a recurring limitation in the EEG-based MCI literature is the way in which generalization capability is evaluated. Many studies segment EEG into multiple epochs and apply standard k-fold cross-validation at the segment level, which can place segments from the same subject in both training and test folds. This setting primarily measures within-subject separability and may yield optimistic performance that does not reflect the clinically relevant scenario, predicting MCI for previously unseen subjects. In contrast, subject-wise (grouped) evaluation and external validation across datasets or paradigms are less frequently reported, even though they are essential for assessing real-world robustness and deployment readiness. Another significant concern is the lack of interpretability; most deep learning models operate as “black boxes,” offering minimal transparency in their decision-making processes, which remains a critical barrier to clinical acceptance. Additionally, EEG-MCI datasets are often skewed with relatively fewer MCI samples, which can bias learning and make accuracy alone less informative, motivating imbalance-aware training procedures and complementary evaluation metrics. Overall, existing models may achieve high classification performance under specific evaluation settings, but they do not consistently address clinically realistic generalization and model explainability, while also incurring considerable computational overhead. These challenges underscore the need for next-generation models that strike a balance between accuracy, efficiency, and transparency. Incorporating spatio-temporal modeling capabilities, attention mechanisms, and explainable artificial intelligence (XAI) techniques is essential for the development of robust, interpretable, and clinically viable EEG-based MCI detection systems.

In this study, we present a novel deep learning framework for the early detection of MCI from EEG signals, with a particular emphasis on protocol-aware generalization capability evaluation and interpretability-oriented model design. The proposed approach targets key challenges in EEG-based MCI detection, including the high dimensionality and spatio-temporal complexity of multichannel EEG, limited sample availability, skewed class distributions, and the limited transparency of many existing deep learning models. Our hybrid architecture integrates one-dimensional CNNs (1D-CNNs) with residual learning and bidirectional recurrent layers (BiLSTM and BiGRU), enabling the extraction of both localized and long-range temporal dependencies in EEG sequences. In addition, Squeeze-and-Excitation (SE) blocks are used to provide adaptive channel-wise recalibration, allowing the network to emphasize clinically informative channels within learned feature representations. To improve robustness without synthetic augmentation, training-fold class weights are computed exclusively from the training split and incorporated during optimization. For clinically meaningful decision-making, predicted probabilities are calibrated using post-hoc temperature scaling, and the operating threshold is selected on validation predictions using Youden’s J statistic to balance sensitivity and specificity; fixed-threshold results (0.35 and 0.50) are also reported to characterize sensitivity-specificity trade-offs. The framework is evaluated using five-fold cross-validation under both subject-dependent (segment-wise) and strict subject-independent (subject-wise) protocols on a primary resting-state segmented EEG dataset, and is further validated on an odor-evoked EEG dataset to strengthen evidence of generalization to unseen subjects. Finally, complementary XAI techniques, including UMAP, SHAP, and LIME, are applied to enhance transparency and support clinical interpretation of the model’s behavior. The main contributions of this work are summarized as follows:

- We conduct a protocol-aware generalization capability evaluation on a primary resting-state EEG dataset using two complementary five-fold settings: segment-wise stratified cross-validation (subject-dependent benchmarking) and subject-wise stratified group cross-validation (strict subject-independent generalization).
- We further perform external subject-independent validation on an odor-evoked EEG dataset to strengthen evidence of generalization to unseen subjects under a controlled paradigm, despite a reduced four-channel montage.
- We propose an end-to-end CNN-Res-SE-BiLSTM-BiGRU architecture that combines convolutional feature extraction, residual learning, and complementary bidirectional recurrent modeling to capture short-range and long-range temporal dynamics in EEG.
- We incorporate SE modules for adaptive channel-wise recalibration, supporting interpretability-oriented design by emphasizing informative EEG channels within learned feature representations.
- We introduce a validation-only clinical decision procedure that applies post-hoc temperature scaling for probability calibration and selects operating thresholds using Youden’s J statistic, with additional fixed-threshold reporting (0.35 and 0.50) to characterize sensitivity–specificity trade-offs.
- We mitigate the effects of skewed labels using fold-specific class weights computed from the training split only, together with binary cross-entropy with label smoothing, avoiding synthetic oversampling while maintaining stable optimization.
- We include baseline comparisons and component-wise ablations under subject-independent evaluation to quantify the contribution of key architectural components.
- We provide multi-level interpretability analysis using UMAP for feature-space visualization and SHAP/LIME for global and instance-level explanations, enabling the identification of influential channel-time patterns and supporting transparent decision tracing.

The remainder of the paper is organized as follows: Sect.  2 describes the EEG dataset, preprocessing pipeline, the proposed CNN-BiLSTM-BiGRU hybrid architecture with SE blocks, training strategies, and evaluation metrics. Section  3 presents the experimental results, including training behavior, classification performance, baseline performance, ablation studies, and model interpretability using UMAP, SHAP, and LIME. Section  4 provides a detailed discussion of the findings and a comparative analysis with existing approaches. Section  5 concludes the paper and outlines potential directions for future research.

## Methodology

This section describes the datasets used, the EEG preprocessing pipeline, the proposed CNN-Res-SE-BiLSTM-BiGRU architecture, the training and calibration procedure, and the evaluation protocols and metrics adopted in this study.

### Primary resting-state EEG dataset

#### Dataset description

This study utilized a publicly available resting-state EEG dataset comprising recordings from 27 subjects recruited from the cardiac units of Sina and Nour Hospitals, Isfahan, Iran [7]. The cohort included 16 cognitively HCs and 11 individuals diagnosed with MCI, aged 60–77 years. Ethical approval for data acquisition was granted by the Deputy of Research and Technology at Isfahan University of Medical Sciences, and informed consent was obtained from all participants. Clinical diagnosis of MCI was based on Petersen’s diagnostic criteria, and the diagnosis was supported by neuropsychological screening using MMSE and NUCOG scores as reported in Table 1. All EEG recordings were performed in the morning while subjects were resting comfortably in a quiet room with their eyes closed to minimize visual interference. EEG activity was acquired continuously for 30 min using a 32-channel digital EEG system (Galileo NT, EBneuro, Italy) at a sampling rate of 256 Hz. Nineteen active electrodes were placed according to the International 10–20 system at Fp1, Fp2, F7, F3, Fz, F4, F8, T3, C3, Cz, C4, T4, T5, P3, Pz, P4, T6, O1, and O2. Electrode-skin impedance was maintained below 5 kΩ throughout the recording. To ensure stable vigilance levels, subjects were continuously monitored to avoid drowsiness, as prolonged rest can induce EEG slowing. Following acquisition, a 50 Hz notch filter was applied to suppress power-line noise, and segments contaminated by motion, electrode displacement, or other non-physiological artifacts were manually excluded through expert visual inspection to ensure high-quality, clean EEG data for analysis. Table 1 summarizes the demographic characteristics of the study participants.

**Table 1:** Demographic characteristics of the dataset

Information	Healthy controls	MCI patients
Age (years)	65.3 ± 3.9	66.4 ± 4.6
Education (years)	11.1 ± 3.0	10.3 ± 3.8
MMSE score	29.0 ± 0.8	27.6 ± 0.9
NUCOG score	91.1 ± 3.0	82.4 ± 3.6

#### EEG data preprocessing

**Fig. 1:**
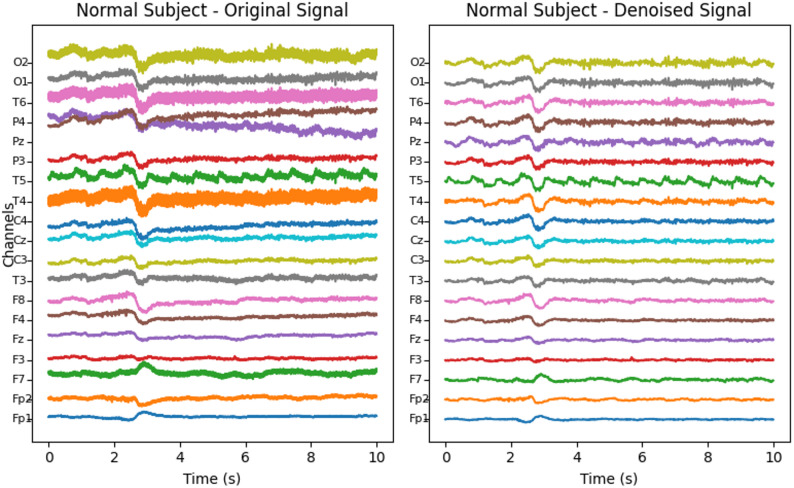
EEG recordings before and after denoising for HC

**Fig. 2:**
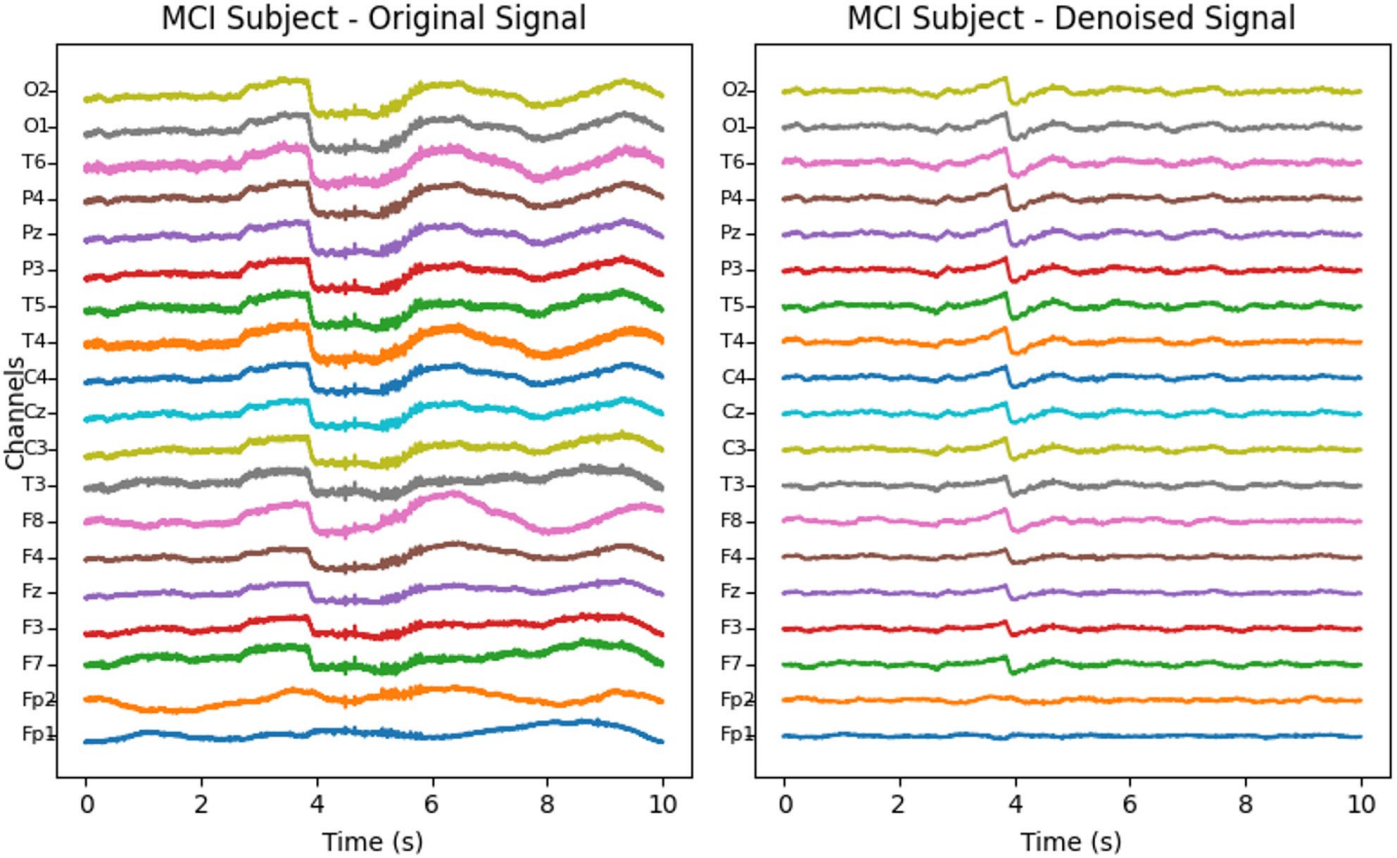
EEG recordings before and after denoising for an MCI patient

The EEG preprocessing pipeline comprised two sequential stages, denoising and segmentation, designed to improve signal quality while preserving diagnostically relevant EEG dynamics for MCI detection. To reduce baseline drift, muscle and ocular artifacts, and 50 Hz power-line interference, signals were first denoised using a two-step filtering procedure: a 50 Hz IIR notch filter to suppress mains noise, followed by a third-order Butterworth band-pass filter with cut-off 0.5–50 Hz to retain the principal EEG rhythms (delta, theta, alpha, beta, and lower gamma) while attenuating low-frequency drift and high-frequency noise; zero-phase forward–backward filtering was applied to avoid phase distortion. The denoised continuous recordings (30 min per subject, 19 channels, 256 Hz; 460,800 samples per channel) were then segmented into non-overlapping 6 s epochs (1,536 samples per segment), yielding 300 segments per subject and 8,100 labeled segments across the 27-subject cohort, with each segment represented as a 19 × 1536 matrix. These preprocessing steps follow the protocol described in [16], and illustrative examples before and after denoising for HC and MCI subjects are shown in Figs. 1 and 2.

### Odor-evoked EEG dataset

In addition to the primary resting-state dataset, we evaluated the proposed framework on a publicly available odor-evoked EEG dataset introduced by Sedghizadeh et al. [22], collected to characterize neural responses to olfactory stimulation in elderly participants with different cognitive statuses and to explore olfactory dysfunction as an early biomarker of neurodegenerative conditions. The dataset comprises recordings from 35 subjects, including 13 with Alzheimer’s disease (AD), 7 with MCI corresponding to amnestic MCI, and 15 HCs, recruited through a memory clinic and screened clinically and neuropsychologically, with exclusion criteria including stroke, traumatic brain injury, and non-AD neurodegenerative disorders. EEG was acquired using a Mitsar amplifier from four electrodes placed according to the international 10–20 system at Fp1, Fz, Cz, and Pz, with Fp1 used to monitor ocular activity. Signals were sampled at 2000 Hz, referenced to the left earlobe, and electrode impedance was maintained below 15 kΩ. Odor stimulation was delivered via a laboratory-grade olfactometer using lemon and rose in a randomized oddball design, where lemon served as the frequent stimulus with 75% probability and rose as the infrequent stimulus with 25% probability; each trial consisted of a 2 s odor presentation followed by an 8 s rest interval.

As reported for this dataset [22], signals were pre-processed using 0.5–40 Hz band-pass filtering, downsampling to 200 Hz, and independent component analysis for artifact removal, and then segmented into epochs from 1 s before to 2 s after stimulus onset, yielding 600 samples per epoch at 200 Hz, with noisy epochs visually screened. For consistency with the MCI detection objective, we adopted a binary classification setting (HC vs. MCI) in which the MCI category corresponds to aMCI. Therefore, only HCs and aMCI subjects were used for training and evaluation, and AD subjects were excluded. We used the provided pre-segmented epochs and applied an additional normalization step consistent with our pipeline: per-epoch, per-channel z-score standardization, in which the mean and SD were computed within each epoch to avoid information leakage across folds.

Collectively, the two datasets provide a complementary evaluation of the proposed framework. The primary dataset contains multichannel resting-state EEG and serves as a benchmark for learning patterns from spontaneous brain activity. The odor-evoked dataset, in contrast, is collected under controlled olfactory stimulation, producing time-locked EEG responses that can be more consistent across trials than unconstrained resting activity. It includes an inter-trial rest interval and a pre-stimulus baseline within each epoch, but does not provide a standalone long-duration resting-state recording comparable to the primary dataset. In addition, the odor dataset uses only four channels, allowing us to assess whether the proposed method remains effective under a reduced and more practical electrode setup.

### Proposed hybrid deep learning architecture

The proposed hybrid deep learning architecture is designed to classify EEG segments into HC and MCI categories by combining convolutional feature extraction with recurrent temporal modeling. The network consists of three main components: (i) a 1D-CNN backbone composed of two convolutional stages with residual connections and squeeze-and-excitation (SE) attention for channel-wise recalibration, (ii) a sequential modeling block formed by a BiLSTM layer followed by layer normalization and a BiGRU layer to capture long-range temporal dependencies, and (iii) a dense classification head that maps the learned temporal representation to a binary sigmoid output. Each input segment is represented as a two-dimensional array of size $$ T\times C$$, where $$ T$$ denotes the number of temporal samples and $$ C$$ denotes the number of EEG channels.

#### CNN backbone

In the proposed model, a 1D convolutional neural network (1D-CNN) is employed to analyze multivariate EEG time-series data [23, 24] and to extract localized temporal features from multiple scalp channels. EEG signals are sequential and multidimensional, with each channel reflecting neural activity from different brain regions. To model these signals, one-dimensional convolutional kernels are applied along the temporal axis, enabling the network to learn discriminative short-range patterns such as transient bursts, rhythmic activity, and localized changes in spectral content. Each EEG segment is provided to the network as a two-dimensional array of size (T × C), where T denotes the number of temporal samples in the segment and C denotes the number of channels. In this formulation, Conv1D operates along the temporal dimension while learning filters that combine information across channels. The CNN backbone comprises two convolutional stages that progressively transform the raw EEG into higher-level representations suitable for downstream temporal modeling and classification.

The first convolutional stage begins with a Conv1D layer containing 64 filters with a kernel size of 3; stride 1, padding “same”, and ReLU activation, followed by batch normalization to stabilize activations and improve convergence. To enhance representational capacity while preserving low-level information, a squeeze-and-excitation (SE) module is then applied to perform channel-wise recalibration. Specifically, global average pooling is used to summarize feature maps, and two fully connected layers (with reduction ratio *r* = 16) learn multiplicative weights that emphasize informative feature maps and suppress less relevant ones [25, 26]. After SE recalibration, a residual block consisting of two Conv1D layers (64 filters, kernel size 3) with batch normalization is applied, and its output is added to the block input through a skip connection, followed by a nonlinearity. This residual learning mechanism improves gradient flow and reduces degradation in deeper feature extraction. Finally, dropout (rate = 0.35) is used for regularization, followed by MaxPooling1D (pool size = 2) to reduce temporal resolution while retaining salient features.

The second convolutional stage follows the same design principles but increases the feature extraction capacity using a Conv1D layer with 128 filters (kernel size 3; stride 1, padding “same”) and ReLU activation, followed by batch normalization, SE recalibration (*r* = 16), and a residual block built with two Conv1D layers (128 filters, kernel size 3) and batch normalization. Dropout (rate = 0.35) and MaxPooling1D (pool size = 2) are again applied at the end of the block. After the second pooling operation, an additional dropout layer (rate = 0.35) is then applied before the recurrent module to further reduce overfitting before long-range temporal modeling. Together, these convolutional stages capture both low-level and higher-level temporal characteristics of EEG signals and produce compact feature sequences that are subsequently passed to the recurrent layers for long-range temporal modeling.

#### BiLSTM

Following the convolutional backbone, the temporal modeling module employs a BiLSTM layer configured with 128 units per direction and return_sequences = True, enabling the network to preserve the full temporal feature sequence for downstream recurrent refinement. Unlike standard unidirectional LSTM layers that process sequences only in a single temporal direction, the BiLSTM architecture processes the input simultaneously from both past-to-future and future-to-past [27–29]. This bidirectional structure enables the model to incorporate both preceding and succeeding temporal context at each time step, which is particularly valuable for EEG signal modeling where relevant patterns may depend on both prior and subsequent neural activity. While the preceding 1D-CNN layers primarily capture short-term and localized temporal features, such as sharp spikes, rhythmic bursts, and localized frequency shifts, the BiLSTM layer is designed to capture longer-range temporal dependencies by integrating information across the entire segment in both directions. To further stabilize learning, layer normalization is applied to the BiLSTM outputs, standardizing activations across hidden units and improving training reliability of the recurrent module. By combining local pattern extraction from the CNN with bidirectional sequential modeling, the architecture captures both fine-grained temporal structure and broader temporal context required for EEG-based classification.

#### BiGRU

Following BiLSTM processing, the temporally enriched features are further refined using a BiGRU layer configured with 64 units per direction. GRUs represent a streamlined variant of LSTM networks, employing reset and update gates to regulate information flow [30]. Unlike LSTMs, GRUs do not maintain a separate cell state; instead, they directly update hidden states, reducing parameter count and computational complexity while retaining the ability to model temporal dependencies effectively. The bidirectional architecture allows the network to integrate context from both past-to-future and future-to-past directions, refining the temporal representations learned by the BiLSTM. In the proposed configuration, the BiGRU summarizes the BiLSTM output sequence into a compact fixed-length representation by aggregating information across time from both directions, providing an efficient refinement stage before classification. By cascading a BiGRU layer after the BiLSTM, the model combines the strong contextual modeling capacity of BiLSTM with the computational efficiency of GRU, enhancing temporal feature representations without substantially increasing complexity. To mitigate overfitting after temporal feature abstraction, a dropout layer with a rate of 0.35 is applied following the BiGRU layer, reducing co-adaptation of units and promoting generalization to unseen EEG data.

#### Dense layers and classification output

**Fig. 3:**
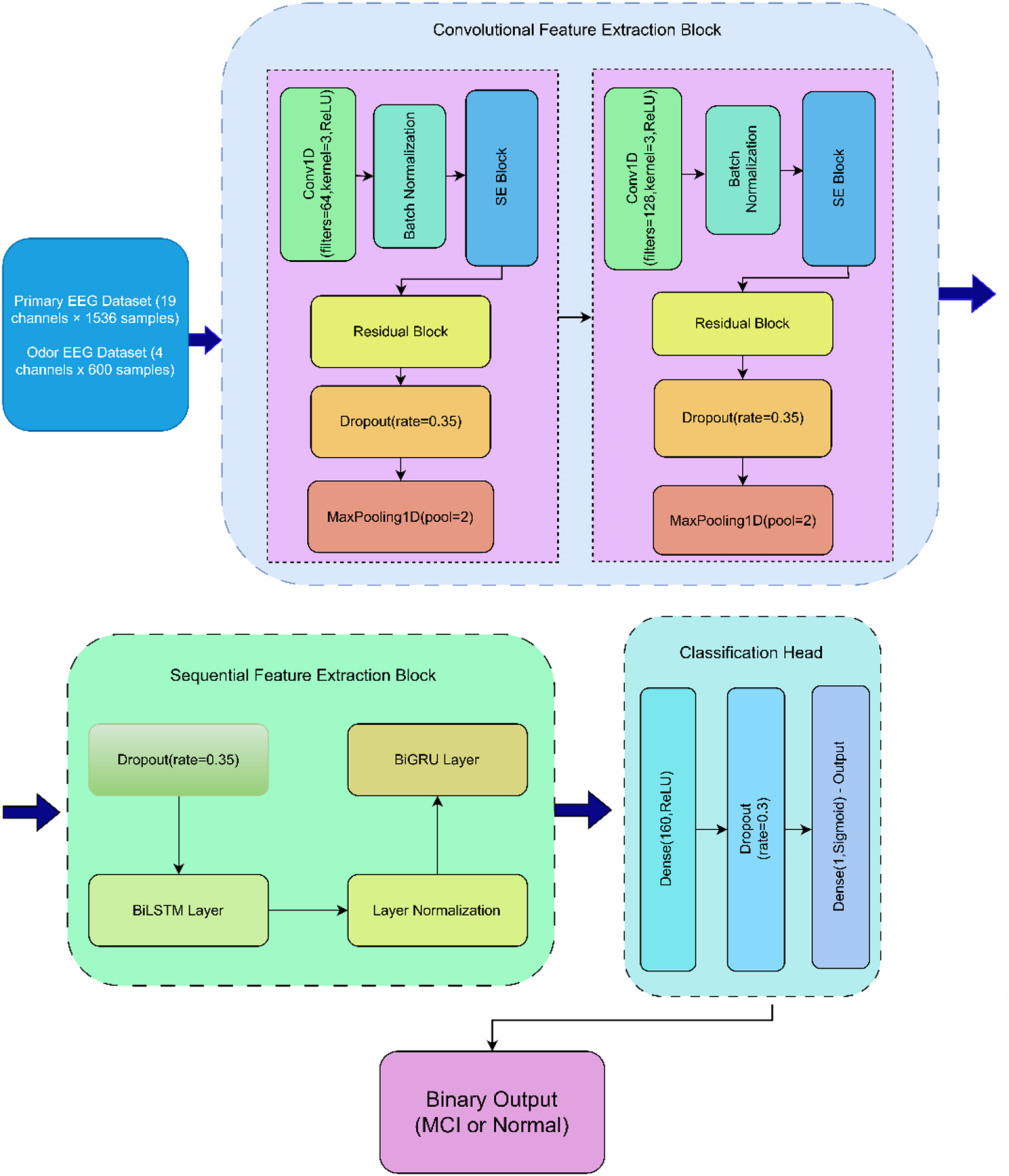
Architecture of the proposed hybrid model

The temporally refined feature representations generated by the recurrent layers are propagated into a fully connected dense layer comprising 160 neurons with ReLU activation, providing an additional stage of high-level feature abstraction before classification. To mitigate overfitting, a dropout layer with a rate of 0.35 is applied after this dense layer. Consistent dropout regularization (rate = 0.35) is also used throughout the network, including after the convolutional stages, prior to the recurrent module, and after the recurrent stack, promoting model robustness and generalization. The output layer consists of a single neuron with sigmoid activation, producing a probability score that indicates the likelihood that the input EEG segment belongs to the MCI class. This configuration is designed for binary classification between HC and MCI. Table 2 summarizes the model hyperparameters, and Fig. 3 provides a schematic overview of the complete architecture.

**Table 2:** Hyperparameters of the hybrid model

Block	Layer/Type	Hyperparameters
Input	Input layer	Primary dataset input shape = (1536, 19) Odor dataset input shape = (600, 4)
CNN block 1	Conv1D + BatchNorm	Filters: 64, Kernel size: 3, Stride: 1, Activation: ReLU, Padding: ‘same’
	SE	Applied after Conv1D + BatchNorm and before the residual block; reduction ratio = 16
	Residual connection	Shortcut connection
	Dropout + MaxPooling	Dropout: 0.35, Pool size: 2
CNN block 2	Conv1D + BatchNorm	Filters: 128, Kernel size: 3, Stride: 1, Activation: ReLU, Padding: ‘same’
	SE	Applied after Conv1D + BatchNorm and before the residual block; reduction ratio = 16
	Residual connection	Shortcut connection
	Dropout + MaxPooling	Dropout: 0.35, Pool size: 2
Pre-RNN	Additional dropout	Dropout = 0.35 applied after CNN Block 2 (post-pooling) before BiLSTM
RNN block	BiLSTM	Units: 128 per direction, Return Sequences: True
	Layer normalization	Applied after BiLSTM
	BiGRU	Units: 64 per direction
	Dropout	Dropout: 0.35
Dense block	Dense layer	Units: 160, Activation: ReLU
	Dropout	Dropout: 0.35
Output	Dense layer	Units: 1, Activation: Sigmoid
Training parameters	Optimizer	Adam, learning rate = 0.001 (default β₁/β₂/ε)
	Loss function	Binary cross-entropy with label smoothing = 0.05
	Batch Size	16
	Max epochs	120
	Validation split	Subject-independent protocol (subject-wise split): StratifiedGroupKFold (inner folds = 4) Segment-wise protocol (segment-wise split): StratifiedKFold (inner folds = 4)
	LR scheduler	ReduceLROnPlateau, Factor = 0.5, Patience = 5
	Early stopping	Patience = 10 (monitor validation loss, restore best weights)
	Class balancing	Training-fold class weights (computed only from training segments)
	Classification threshold	Fixed threshold and validation-only Youden’s J (fold-specific)
	Random seed	42

#### Training strategy and model optimization

The performance evaluation of the proposed model was conducted using two complementary stratified five-fold cross-validation protocols to assess performance at both the segment and subject granularity. First, a segment-wise stratified five-fold cross-validation protocol was employed, where individual EEG segments were treated as samples and stratified across folds to preserve class proportions. In each iteration, four folds were used for training and the remaining fold was used for testing. A validation subset was derived exclusively from the training portion using a stratified split and was used for early stopping, probability calibration, and decision-threshold selection. This protocol evaluates performance under a segment-level operating scenario, quantifying how consistently the model separates HC and MCI segments when the evaluation unit is an EEG epoch/segment. Second, a subject-wise stratified group five-fold cross-validation protocol was employed, where the dataset was partitioned into five folds at the subject level while maintaining the HC/MCI distribution across folds. In each iteration, entire subjects were held out as the independent test fold, and the remaining subjects formed the training pool. From this training pool, a subject-wise validation split was further created and used exclusively for early stopping, probability calibration, and decision-threshold selection, ensuring that all segments from a given subject remained confined to a single split (train/validation/test) within a fold. This protocol evaluates the model’s ability to generalize to previously unseen subjects, which is important for clinical deployment.

Operating thresholds were evaluated using both fixed and validation-tuned strategies. Specifically, fixed decision thresholds of 0.35 and 0.50 were considered to examine sensitivity–specificity trade-offs, with the 0.35 threshold intended to improve sensitivity to the minority MCI class relative to the conventional 0.5 threshold. In addition, validation predictions were calibrated using post-hoc temperature scaling. Given logits $$ z$$, calibrated probabilities were computed as $$ p=\sigma (z/T)$$, where the scalar $$ T>0$$ was estimated on the validation split by minimizing the negative log-likelihood. Following calibration, Youden’s J statistic (computed as sensitivity + specificity − 1) was used to determine the operating threshold that maximizes the combined true positive and true negative rates. Importantly, temperature scaling and threshold selection were performed using validation predictions only, and the selected operating point was applied unchanged to the held-out test fold, ensuring that test data were not used for calibration or threshold determination.

The final model optimization employed the Adam optimizer with a learning rate of 0.001, and training was conducted for a maximum of 120 epochs with a batch size of 16. Multiple regularization and monitoring strategies were implemented to ensure stable convergence and to mitigate overfitting: (i) early stopping was applied if validation loss did not improve for 10 consecutive epochs, with restoration of the best weights; (ii) learning rate reduction on plateau was used, reducing the learning rate by a factor of 0.5 upon stagnation of validation loss (patience = 5); (iii) dropout regularization was incorporated across convolutional, recurrent, and dense layers; and (iv) training-fold class weights were computed from the training split only and used during optimization to mitigate class imbalance without synthetic augmentation. Overall, this training and evaluation framework combines validation-only calibration and threshold selection with subject-wise testing to support robust generalization assessment on unseen EEG subjects, while also providing a segment-level benchmarking protocol for comparison with prior work.

#### Evaluation protocol and performance metrics

Following training, the optimal model obtained from each fold was evaluated on its corresponding test set using a comprehensive suite of performance metrics, including Accuracy, Sensitivity (Recall), Specificity, Precision, F1-score, Balanced accuracy, Receiver Operating Characteristic Area Under the Curve (ROC-AUC), and Precision-Recall Area Under the Curve (PR-AUC). Confusion matrices from all folds were aggregated to provide a holistic overview of classification performance. In addition, ROC and precision-recall curves were generated per fold to visualize classification behavior across varying thresholds and to assess the model’s discriminative capability. The computation of these metrics followed their standard mathematical definitions, as outlined below:

*Accuracy* measures the overall correctness of predictions:

$$ Accuracy = \frac{{TP + TN}}{{TP + TN + FP + FN}} $$

where *TP*, *TN*, *FP*, and *FN* are true positives, true negatives, false positives, and false negatives, respectively.

*Sensitivity* indicates the proportion of correctly identified MCI cases:

$$ Sensitivity = \frac{{TP}}{{TP + FN}} $$

*Specificity* reflects correctly identified HCs:

$$ Specificity = \frac{{TN}}{{TN + FP}} $$

*Precision* represents the proportion of correct positive predictions:

$$ Precision = \frac{{TP}}{{TP + FP}} $$

*F1-score* represents the harmonic mean of precision and sensitivity, providing a balanced metric in the presence of class imbalance:

$$ F_{1} = 2 \times \frac{{Precision \times Sensitivity}}{{Precision + Sensitivity}} $$

*Balanced accuracy* provides a fair assessment of the model’s ability to correctly identify both MCI patients and HCs, particularly in the presence of class imbalance where MCI cases are underrepresented.

$$ Balanced\;accuracy~ = ~\frac{{{\mathrm{Sensitivity}} + {\mathrm{Specificity}}}}{2} $$

*ROC-AUC* measures the model’s ability to distinguish between MCI and HCs across all classification thresholds. Higher values indicate better overall discriminative performance.

*PR-AUC*: Focuses on the balance between precision and recall, highlighting the model’s effectiveness in correctly identifying MCI cases in imbalanced datasets. Higher values reflect stronger minority class detection.

All training and evaluation experiments were conducted on a system equipped with an Intel Core i5-12500 H processor, 16 GB DDR4 RAM, and an NVIDIA GeForce RTX 3050 Laptop GPU with 4 GB dedicated memory.

## Results

This section reports the performance of the proposed CNN-Res-SE-BiLSTM-BiGRU model on the primary EEG dataset and the odor EEG dataset. All results follow the training, calibration, and threshold-selection framework described in Sect.  2.3.5, including stratified five-fold cross-validation with validation-only early stopping, probability calibration via temperature scaling, and operating-threshold selection using either validation Youden’s J or fixed thresholds (0.35 and 0.50). For the primary dataset, results are presented under two complementary protocols, segment-wise stratified cross-validation (subject-dependent) and subject-wise stratified group cross-validation (subject-independent), to distinguish segment-level separability from generalization to unseen subjects. The odor EEG dataset is then evaluated under a strict subject-independent protocol to provide a more reliable assessment of subject-independent generalization, followed by pooled confusion-matrix analysis, ROC and PR curve evaluation, and comparative studies including baseline and ablation analyses. Unless otherwise stated, all metrics are reported as mean ± SD across folds.

### Results on primary dataset

#### Subject-dependent evaluation (segment-wise stratified five-fold cross-validation)

In the subject-dependent protocol, EEG segments were treated as independent samples and stratified across folds to preserve class proportions. This setting quantifies how consistently the model separates HC and MCI segments when the evaluation unit is an EEG epoch/segment. Under validation-selected Youden’s J thresholding, the model achieved strong segment-level performance (Table 3), with accuracy 0.974 ± 0.022 and balanced accuracy 0.973 ± 0.022. Sensitivity was 0.963 ± 0.036, and specificity was 0.984 ± 0.016, with precision 1.000 ± 0.000 and F1-score 0.981 ± 0.019. Threshold-independent discrimination was markedly high in this setting (ROC-AUC 0.999 ± 0.001; PR-AUC 1.000 ± 0.000). The validation-selected operating threshold was 0.758 ± 0.110, and temperature scaling produced a small calibration temperature (0.200 ± 0.000), consistent with highly confident and well-separated predictions under this protocol. Fixed-threshold operation produced similarly strong results. At threshold 0.35, accuracy was 0.999 ± 0.001 and balanced accuracy was 0.999 ± 0.001, with sensitivity 1.000 ± 0.001 and specificity 0.998 ± 0.002. At threshold 0.50, performance was essentially identical (accuracy 0.999 ± 0.001; balanced accuracy 0.999 ± 0.001; sensitivity 1.000 ± 0.001; specificity 0.998 ± 0.002). These results indicate that in the segment-wise protocol, the model’s class separation is sufficiently strong that modest changes in the operating point do not materially affect outcomes. As expected, ROC-AUC and PR-AUC remain unchanged across operating thresholds because they are computed by sweeping the threshold. The strong segment-level scores confirm that the proposed architecture is highly effective at separating HC and MCI segments when training and testing data come from the same pool of subjects. However, this protocol is intrinsically subject-dependent: segments from the same individual can be present in both training and testing folds, and subject-specific signal characteristics or recording conditions may inadvertently be exploited by the model. Consequently, strong segment-wise performance should be interpreted primarily as a benchmarking result rather than definitive evidence of generalization to unseen subjects.

**Table 3:** Primary dataset: Segment-wise 5-fold results of the proposed model under different thresholding strategies

Metric	Youden (val)	Fixed 0.35	Fixed 0.50
Accuracy	0.974 ± 0.022	0.999 ± 0.001	0.999 ± 0.001
Balanced accuracy	0.973 ± 0.022	0.999 ± 0.001	0.999 ± 0.001
Sensitivity	0.963 ± 0.036	1.000 ± 0.001	1.000 ± 0.001
Specificity	0.984 ± 0.016	0.998 ± 0.002	0.998 ± 0.002
Precision	1.000 ± 0.000	0.999 ± 0.001	0.999 ± 0.001
F1-score	0.981 ± 0.019	0.999 ± 0.001	0.999 ± 0.001
ROC-AUC	0.999 ± 0.001	0.999 ± 0.001	0.999 ± 0.001
PR-AUC	1.000 ± 0.000	1.000 ± 0.000	1.000 ± 0.000
Operating threshold	0.758 ± 0.110	0.350 ± 0.000	0.500 ± 0.000
Temperature T	0.200 ± 0.000	0.200 ± 0.000	0.200 ± 0.000

#### Subject-independent evaluation (subject-wise stratified group five-fold cross-validation)

To assess generalization to unseen individuals, we additionally employed a subject-wise stratified group five-fold protocol, where all segments from a given subject were confined to a single split within a fold. This setting is more representative of clinical deployment, where the model must generalize to new patients. In this strict subject-independent setting, performance decreased substantially, and variability increased (Table 4). Using validation-selected Youden’s J, accuracy was 0.560 ± 0.161, and balanced accuracy was 0.549 ± 0.220. Sensitivity and specificity were 0.541 ± 0.486 and 0.556 ± 0.321, respectively, with a precision of 0.505 ± 0.341 and an F1-score of 0.416 ± 0.337. Discriminative performance was modest (ROC-AUC 0.574 ± 0.310; PR-AUC 0.546 ± 0.280), indicating limited intrinsic separability when evaluated on unseen subjects. The validation-selected operating threshold was 0.568 ± 0.165. Temperature scaling produced substantially larger temperatures (4.222 ± 1.143), consistent with over-confident raw probabilities that require stronger calibration under subject-independent evaluation. Fixed thresholds revealed the expected sensitivity–specificity trade-off. A fixed threshold of 0.35 increased sensitivity to 0.648 ± 0.435 but reduced specificity sharply to 0.282 ± 0.284, resulting in an accuracy of 0.484 ± 0.158 and balanced accuracy of 0.465 ± 0.221. A fixed threshold of 0.50 improved specificity to 0.420 ± 0.223 but reduced sensitivity to 0.516 ± 0.496, producing an accuracy of 0.482 ± 0.211 and balanced accuracy of 0.468 ± 0.260. Overall, Youden’s J provided the most balanced operating point on average, but the high standard deviations indicate that performance remains fold-dependent and unstable. The subject-independent results highlight a key limitation of the primary dataset: with only 27 subjects, each fold contains a small number of held-out subjects and an even smaller number of validation subjects for calibration and threshold selection. This amplifies fold-to-fold variability and makes operating-point estimation less stable. Moreover, the reduction in ROC-AUC and PR-AUC relative to the segment-wise protocol indicates that the discriminative patterns learned under subject-dependent splitting do not transfer reliably to unseen subjects in this cohort. Therefore, although the primary dataset is suitable for segment-level benchmarking, it provides a limited basis for drawing strong conclusions about subject-independent generalization.

Taken together, the primary dataset results demonstrate very strong segment-level discrimination under the subject-dependent protocol, but substantially weaker and unstable performance under strict subject-wise evaluation, reflecting inter-subject heterogeneity and the small number of subjects available per fold. To provide a stronger subject-independent validation under a controlled paradigm, we next evaluate the same architecture on the odor-evoked EEG dataset, where stimulus-locked responses are expected to yield more consistent subject-independent signatures than spontaneous resting-state activity.

**Table 4:** Primary dataset: Subject-independent 5-fold results of the proposed model under different thresholding strategies

Metric	Youden’s J on validation	Fixed threshold 0.35	Fixed threshold 0.50
Accuracy	0.560 ± 0.161	0.484 ± 0.158	0.482 ± 0.211
Balanced accuracy	0.549 ± 0.220	0.465 ± 0.221	0.468 ± 0.260
Sensitivity	0.541 ± 0.486	0.648 ± 0.435	0.516 ± 0.496
Specificity	0.556 ± 0.321	0.282 ± 0.284	0.420 ± 0.223
Precision	0.505 ± 0.341	0.393 ± 0.250	0.319 ± 0.296
F1-score	0.416 ± 0.337	0.464 ± 0.290	0.379 ± 0.353
ROC-AUC	0.574 ± 0.310	0.574 ± 0.310	0.574 ± 0.310
PR-AUC	0.546 ± 0.280	0.546 ± 0.280	0.546 ± 0.280
Operating threshold	0.568 ± 0.165	0.350 ± 0.000	0.500 ± 0.000
Temperature T	4.222 ± 1.143	4.222 ± 1.143	4.222 ± 1.143

### Results on the odor EEG dataset

Following the common evaluation procedure described above, the odor EEG dataset was evaluated using subject-independent five-fold cross-validation with validation-only early stopping, temperature scaling, and operating-threshold selection.

#### Subject-independent evaluation (subject-wise stratified five-fold cross-validation)

Table 5 summarizes performance under different operating-threshold strategies on temperature-calibrated probabilities. Youden-based thresholding achieved the strongest overall threshold-dependent performance, with accuracy 0.956 ± 0.051 and balanced accuracy 0.954 ± 0.043. At the selected operating threshold of 0.476 ± 0.359, the model achieved a sensitivity of 0.965 ± 0.021 and a specificity of 0.943 ± 0.093, indicating that false negatives are kept low while false positives are controlled. Fixed-threshold operation produced comparable performance and showed the expected trade-off: threshold 0.35 slightly increased sensitivity (0.966 ± 0.022) while reducing specificity (0.929 ± 0.134), whereas threshold 0.50 reduced sensitivity (0.961 ± 0.024) with a small specificity increase relative to 0.35 (0.931 ± 0.131); however, it remained below the Youden operating point. As expected, ROC-AUC (0.971 ± 0.051) and PR-AUC (0.934 ± 0.132) were unchanged across threshold strategies. The temperature estimate was identical across the three threshold strategies (T = 0.561 ± 0.385), because calibration was learned once on the validation split and only the final decision threshold was altered.

**Table 5:** Odor EEG dataset: Subject-independent 5-fold results of the proposed model under different thresholding strategies

Metric	Youden’s J on validation	Fixed threshold 0.35	Fixed threshold 0.50
Accuracy	0.956 ± 0.051	0.949 ± 0.072	0.949 ± 0.071
Balanced accuracy	0.954 ± 0.043	0.947 ± 0.060	0.946 ± 0.059
Sensitivity	0.965 ± 0.021	0.966 ± 0.022	0.961 ± 0.024
Specificity	0.943 ± 0.093	0.929 ± 0.134	0.931 ± 0.131
Precision	0.936 ± 0.093	0.930 ± 0.124	0.931 ± 0.122
F1-score	0.948 ± 0.047	0.943 ± 0.065	0.942 ± 0.063
ROC-AUC	0.971 ± 0.051	0.971 ± 0.051	0.971 ± 0.051
PR-AUC	0.934 ± 0.132	0.934 ± 0.132	0.934 ± 0.132
Operating threshold	0.476 ± 0.359	0.350 ± 0.000	0.500 ± 0.000
Temperature (T)	0.561 ± 0.385	0.561 ± 0.385	0.561 ± 0.385

**Fig. 4:**
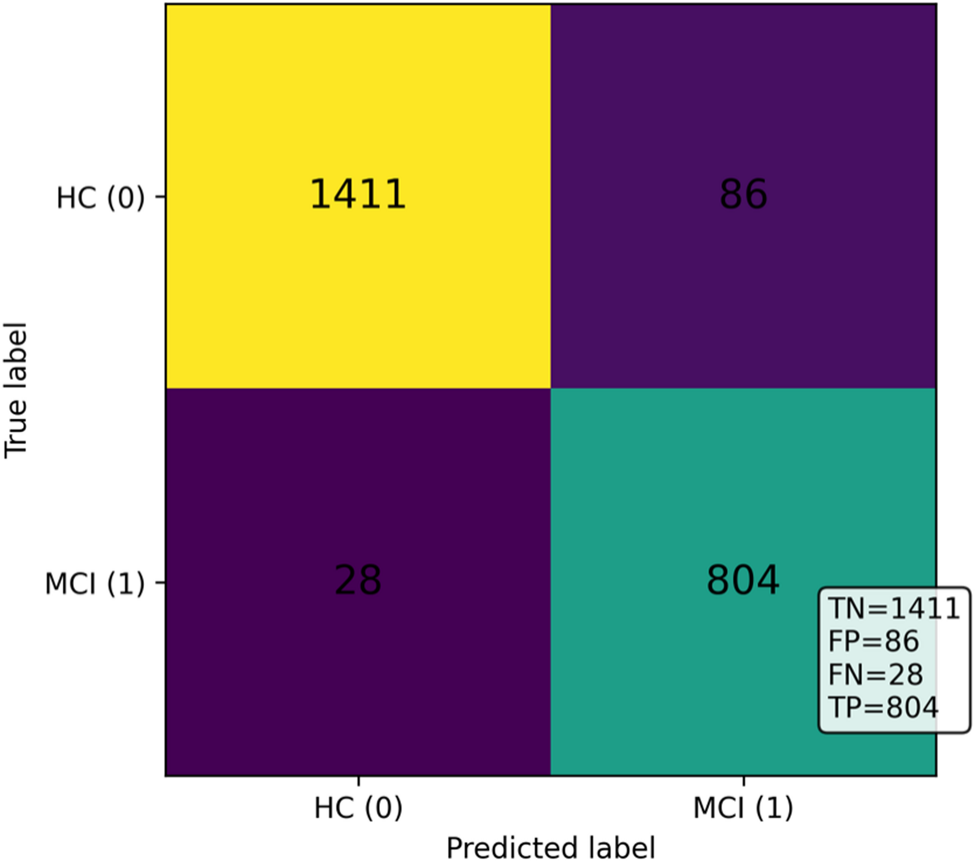
Pooled segment-level confusion matrix for the subject-independent odor-evoked EEG dataset

**Fig. 5:**
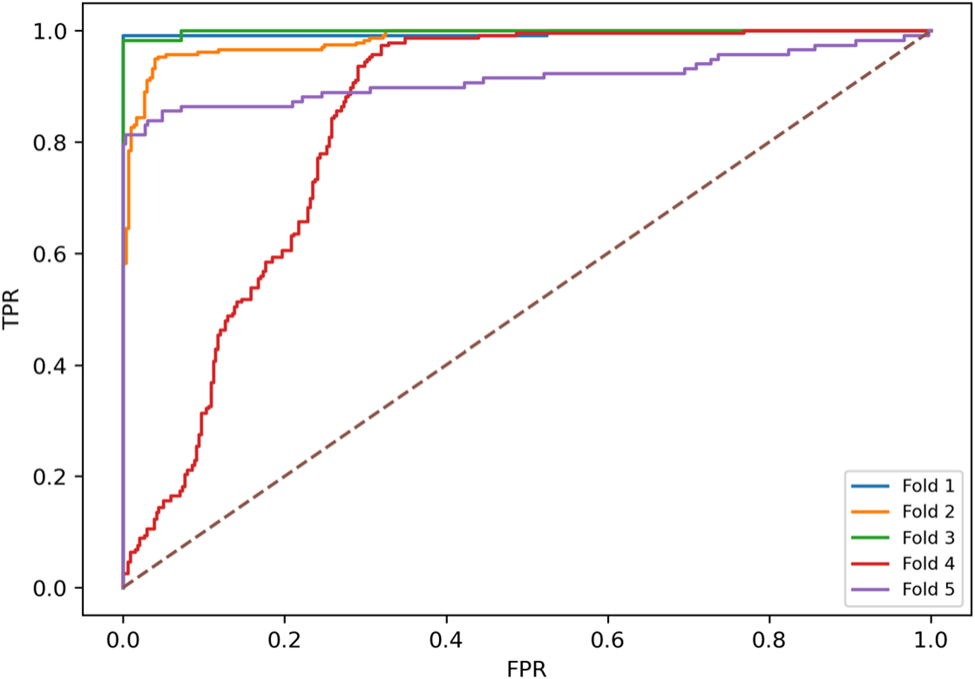
ROC curves across folds for subject-independent MCI classification on the odor-evoked EEG

**Fig. 6:**
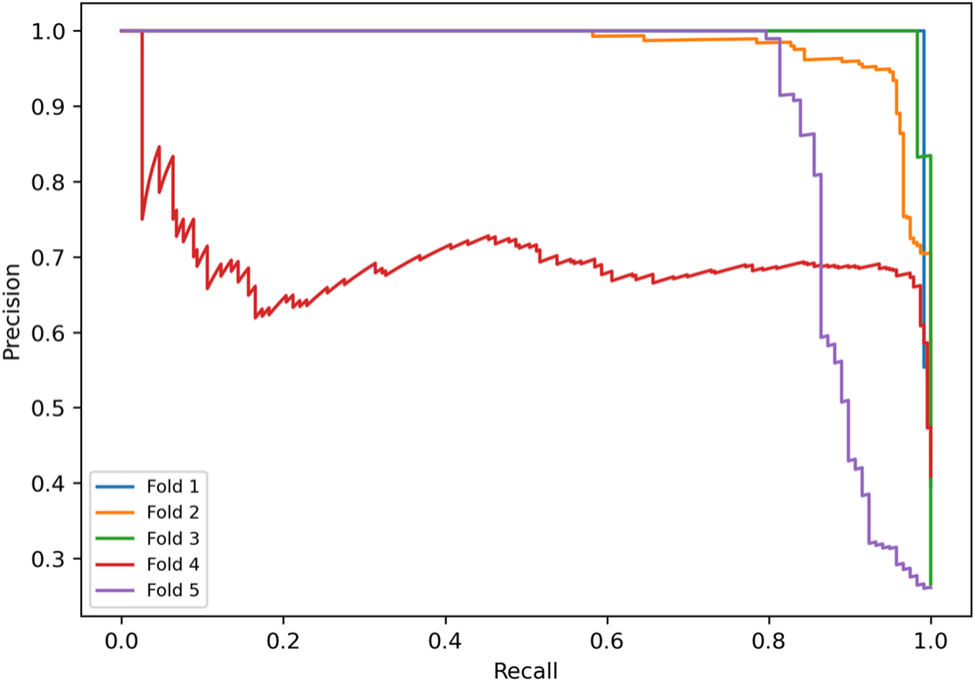
Precision–recall curves across folds for subject-independent MCI classification on the odor-evoked EEG dataset

Further, to complement the operating-point metrics in Table 5, we report pooled confusion-matrix counts and threshold-independent ROC and PR curves to characterize the error structure and the stability of discrimination across folds under subject-independent evaluation. Figure 4 shows the pooled segment-level confusion matrix of the proposed model under the subject-independent five-fold protocol using the validation-selected Youden operating point after temperature scaling. When aggregating test predictions across folds, the pooled confusion matrix yielded TN = 1411, FP = 86, FN = 28, and TP = 804, reflecting a low false-negative count alongside controlled false positives. These pooled counts correspond to a pooled sensitivity of 804/(804 + 28) = 0.966 and a pooled specificity of 1411/(1411 + 86) = 0.943. Small differences between these pooled values and Table 5 are expected because Table 5 reports mean ± SD across folds, whereas Fig. 4 reflects a pooled aggregation across all folds. Figure 5 presents the ROC curves obtained from the five cross-validation folds under the same protocol. The curves show consistently high true-positive rates across a broad range of false-positive rates, indicating strong discrimination and limited fold-to-fold variation, which is consistent with the ROC-AUC of 0.971 ± 0.051 reported in Table 5. Figure 6 illustrates the corresponding PR curves for the same folds. Despite moderate class imbalance, the curves remain tightly clustered, indicating stable precision across varying recall levels. The PR-AUC is 0.934 ± 0.132 as reported in Table 5, and at the selected operating point Table 5 reports a mean precision of 0.936 ± 0.093 and a mean F1-score of 0.948 ± 0.047 across folds.

#### Baseline model comparison

We compared the proposed approach against representative baseline architectures, including single-stream models, CNN-only, LSTM-only, and hybrid CNN–recurrent variants (Table 6). All baseline models were evaluated under the same subject-independent protocol with validation-only temperature scaling and validation-selected Youden thresholding. These results show that combining convolutional feature extraction with bidirectional recurrent modeling generally improves performance relative to purely recurrent processing, and that the choice of recurrent head influences both the operating-point metrics and the overall separability captured by AUC measures. Among the baselines, CNN+BiLSTM + GRU-head achieved the strongest overall performance, with accuracy 0.934 ± 0.074 and balanced accuracy of 0.936 ± 0.056. It also maintained a favorable trade-off between sensitivity (0.966 ± 0.033) and specificity (0.906 ± 0.132), resulting in a high F1-score (0.925 ± 0.070). CNN+LSTM (Accuracy 0.913 ± 0.074) and CNN + GRU (Accuracy 0.900 ± 0.077) were competitive but slightly weaker. In contrast, LSTM-only yielded the lowest operating-point performance (accuracy 0.862 ± 0.125; balanced accuracy 0.847 ± 0.115). The ranking-based metrics further highlight these differences, with ROC-AUC ranging from 0.934 ± 0.051 to 0.996 ± 0.002 and PR-AUC ranging from 0.880 ± 0.087 to 0.996 ± 0.002. The calibration parameters also vary across baselines (temperature $$ T$$ and Youden threshold), reflecting that different architectures produce different confidence distributions and therefore require different degrees of calibration and different operating points under subject-independent evaluation.

**Table 6:** Odor EEG dataset: Baseline models comparison under subject-independent 5-fold evaluation

Metric	CNN-only	LSTM-only	CNN+LSTM	CNN + GRU	CNN+BiLSTM + GRU-head
Accuracy	0.894 ± 0.071	0.862 ± 0.125	0.913 ± 0.074	0.900 ± 0.077	0.934 ± 0.074
Balanced accuracy	0.894 ± 0.052	0.847 ± 0.115	0.893 ± 0.096	0.876 ± 0.117	0.936 ± 0.056
Sensitivity	0.943 ± 0.097	0.870 ± 0.175	0.868 ± 0.192	0.860 ± 0.264	0.966 ± 0.033
Specificity	0.845 ± 0.147	0.824 ± 0.252	0.918 ± 0.110	0.892 ± 0.145	0.906 ± 0.132
Precision	0.840 ± 0.149	0.805 ± 0.149	0.904 ± 0.125	0.888 ± 0.134	0.898 ± 0.135
F1-score	0.876 ± 0.062	0.820 ± 0.118	0.869 ± 0.123	0.840 ± 0.168	0.925 ± 0.070
ROC-AUC	0.987 ± 0.020	0.934 ± 0.051	0.962 ± 0.037	0.994 ± 0.003	0.996 ± 0.002
PR-AUC	0.981 ± 0.033	0.880 ± 0.087	0.942 ± 0.056	0.991 ± 0.006	0.996 ± 0.002
Temperature (T)	0.377 ± 0.159	1.248 ± 0.756	0.487 ± 0.306	0.551 ± 0.334	0.440 ± 0.218
Youden threshold	0.726 ± 0.398	0.510 ± 0.346	0.515 ± 0.452	0.771 ± 0.421	0.578 ± 0.413

#### Ablation studies

Table 7 summarizes an ablation study to quantify the contribution of key components in the proposed architecture. This study evaluates the contribution of residual learning, SE channel recalibration, and bidirectional recurrent components by removing one element at a time while keeping the training and evaluation protocol unchanged. The full model achieved an accuracy of 0.956 ± 0.051, balanced accuracy of 0.954 ± 0.043, and an F1-score of 0.948 ± 0.047. Removing individual components consistently reduced performance, indicating that the final accuracy is obtained through the combined effect of residual learning, channel recalibration, and bidirectional temporal modeling. Removing the SE block reduced accuracy to 0.922 ± 0.078 and balanced accuracy to 0.917 ± 0.065, indicating that channel-wise recalibration helps the network emphasize informative feature maps. Removing residual connections produced a comparable reduction (accuracy 0.916 ± 0.076; balanced accuracy 0.909 ± 0.067), consistent with the role of residual learning in stabilizing optimization and preserving discriminative representations. Removing BiLSTM reduced accuracy to 0.895 ± 0.079 and lowered precision to 0.837 ± 0.150 and F1-score to 0.881 ± 0.070, suggesting that bidirectional long-range context is important for maintaining a strong precision–recall balance. The largest degradation was observed when removing BiGRU. In this setting, balanced accuracy decreased to 0.861 ± 0.158, and the threshold-independent metrics dropped sharply (ROC-AUC 0.854 ± 0.259; PR-AUC 0.851 ± 0.242). This behavior indicates that the BiGRU component is critical for preserving class separability across unseen subjects, rather than merely shifting the operating point. In addition, the operating thresholds and temperatures vary markedly across ablations (Table 7), showing that removing components changes the distribution of predicted confidences and consequently affects both calibration strength and the threshold that optimizes the sensitivity-specificity balance.

**Table 7:** Odor EEG dataset: ablation study of the proposed model under subject-independent 5-fold evaluation

Metric	Proposed (full)	w/o SE	w/o residual	w/o BiLSTM	w/o BiGRU
Accuracy	0.956 ± 0.051	0.922 ± 0.078	0.916 ± 0.076	0.895 ± 0.079	0.893 ± 0.098
Balanced accuracy	0.954 ± 0.043	0.917 ± 0.065	0.909 ± 0.067	0.899 ± 0.058	0.861 ± 0.158
Sensitivity	0.965 ± 0.021	0.938 ± 0.080	0.924 ± 0.116	0.951 ± 0.059	0.828 ± 0.345
Specificity	0.943 ± 0.093	0.897 ± 0.141	0.894 ± 0.145	0.847 ± 0.147	0.895 ± 0.152
Precision	0.936 ± 0.093	0.894 ± 0.146	0.881 ± 0.132	0.837 ± 0.150	0.894 ± 0.150
F1-score	0.948 ± 0.047	0.906 ± 0.076	0.890 ± 0.073	0.881 ± 0.070	0.802 ± 0.264
ROC-AUC	0.971 ± 0.051	0.960 ± 0.036	0.965 ± 0.044	0.973 ± 0.039	0.854 ± 0.259
PR-AUC	0.934 ± 0.132	0.919 ± 0.098	0.944 ± 0.054	0.960 ± 0.063	0.851 ± 0.242
Temperature	0.561 ± 0.385	0.439 ± 0.241	0.525 ± 0.245	0.469 ± 0.223	0.556 ± 0.228
Youden threshold	0.476 ± 0.359	0.863 ± 0.182	0.517 ± 0.397	0.522 ± 0.441	0.305 ± 0.401

In summary, the odor-evoked EEG dataset demonstrates strong and stable subject-independent performance for the proposed framework, supported by operating-point evaluation, pooled error analysis, and consistent ROC and PR behavior across folds. Baseline comparisons and ablation studies further confirm that the proposed architectural components contribute meaningfully to this performance under the same evaluation protocol.

### Model explainability and interpretability

To better understand the decision-making process of the proposed CNN-Res-SE-BiLSTM-BiGRU model for MCI classification, we incorporated multiple interpretability techniques. UMAP was used to visualize the learned feature space and assess class separability, SHAP provided global feature attributions identifying the most influential electrode–time-bin features driving the model output, and LIME offered local, instance-level explanations for representative segment predictions. These interpretability analyses are reported for the primary dataset under the segment-wise subject-dependent setting, since this protocol preserves the full multichannel electrode configuration and yields stable segment-level separation to support meaningful electrode-wise and segment-level analysis. Together, these methods provide complementary qualitative insight into the model’s learned representations and decision logic at both the cohort level and for representative individual predictions.

#### UMAP-based feature space visualization

**Fig. 7:**
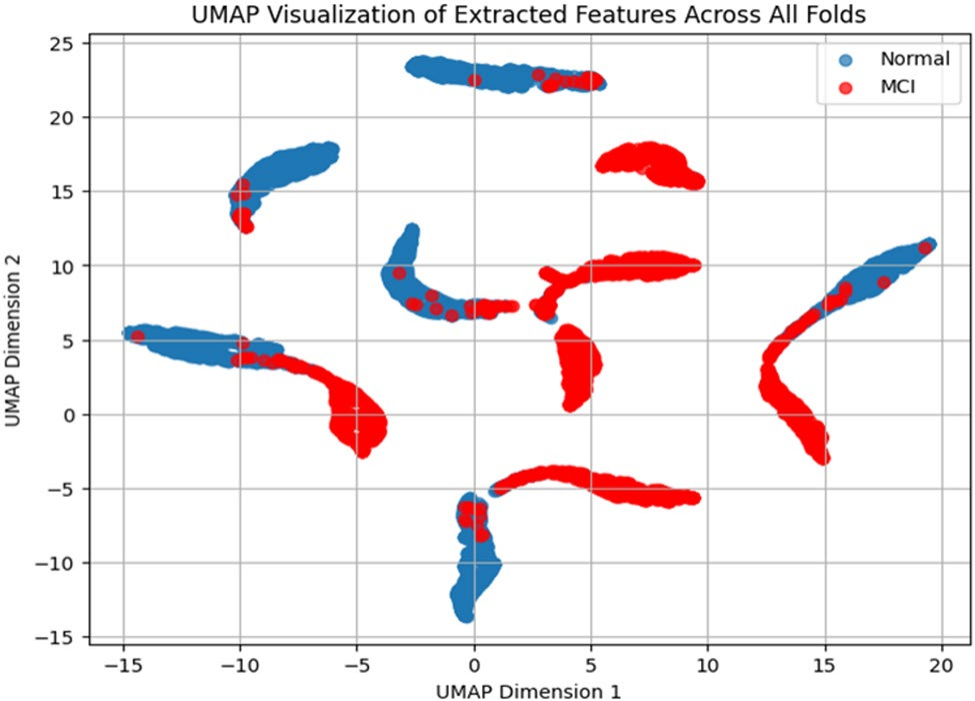
UMAP projection of learned feature embeddings on the primary dataset (segment-wise setting), showing separation patterns between HC and MCI classes across folds

UMAP was used to reduce the high-dimensional feature vectors into a two-dimensional space. These feature vectors were combined from all cross-validation folds. This reduction made it easier to analyze how well the data points from different classes could be separated and how they were distributed. UMAP is effective at preserving both the local and global structure of the original data, which helps in creating a meaningful visualization of complex data patterns [31, 32]. The resulting plot, shown in Fig. 7, displays data points with different colors based on their class. Blue points represent the HC group, and red points represent the MCI group. The two-dimensional plot shows that the two classes form somewhat separate clusters. However, there are areas where the points from the two groups overlap. This overlap suggests that the features of some individuals in the HC and MCI groups are similar. This visual result is supported by a Silhouette Coefficient of 0.6452. The Silhouette Coefficient is a measure used to evaluate the quality of clustering [33, 34]. It ranges from − 1 to 1, where higher values indicate better-defined and more clearly separated clusters. A value close to 1 means that the data points are well matched to their cluster and poorly matched to neighboring clusters. A value near 0 suggests overlapping clusters, and negative values indicate that data points may have been assigned to the wrong cluster. In this case, the value of 0.6452 suggests a moderate level of separation and compactness between the two groups. The presence of overlapping areas in the plot highlights the fact that the features extracted are only partially effective in separating the two groups. It also reflects the challenge of distinguishing between normal individuals and those with MCI, which is often difficult due to the subtle nature of early cognitive decline.

#### SHAP analysis

**Fig. 8:**
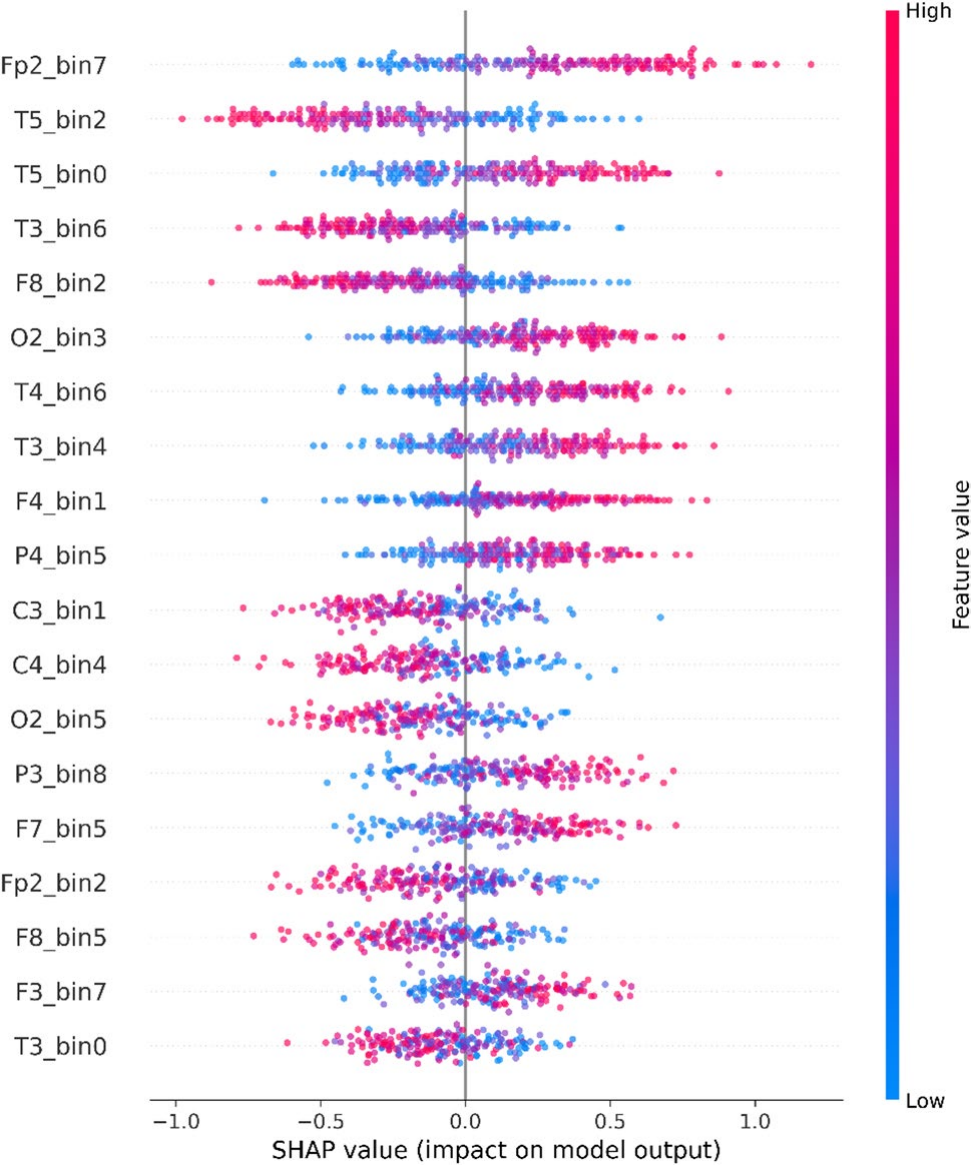
SHAP beeswarm plot of the top electrode-time-bin features for MCI vs. HC classification (primary dataset, segment-wise setting); color denotes feature value and the x-axis shows SHAP impact on the model output

This SHAP [35, 36] summary, depicted in Fig. 8, provides a clear visualization of the features that most strongly influence the classification model in distinguishing individuals with MCI from HCs using EEG data. Each feature is denoted by a composite label consisting of the electrode name and a time-bin index (e.g., T5_bin2, Fp2_bin7). These labels represent signal activity at spatially defined electrodes and within discrete temporal segments obtained after signal segmentation and downsampling. In this context, the term bin refers exclusively to the temporal index within the segmented EEG window and does not correspond to a frequency band. Electrode names follow the international 10–20 system and correspond to specific cortical regions, such as T5 (left temporal), Fp2 (right prefrontal), O2 (right occipital), and T3 (left temporal), thereby preserving anatomical interpretability. In the SHAP visualization, the color gradient encodes the feature value: red indicates higher values within the specified electrode–time bin, whereas blue indicates lower values.

Among the highest-impact features in Fig. 8 are Fp2_bin7, T5_bin2, T5_bin0, T3_bin6, F8_bin2, and O2_bin3, followed by contributions from T4_bin6, T3_bin4, F4_bin1, P4_bin5, C3_bin1, C4_bin4, O2_bin5, P3_bin8, F7_bin5, Fp2_bin2, F8_bin5, F3_bin7, and T3_bin0. For example, Fp2_bin7 shows predominantly positive SHAP values when the feature value is high (red points), indicating that higher values in this prefrontal electrode–time bin shift the model output toward MCI. A similar pattern is observed for T5_bin0, O2_bin3, T4_bin6, T3_bin4, F4_bin1, P4_bin5, P3_bin8, F7_bin5, and F3_bin7, where higher feature values are generally associated with positive SHAP contributions. In contrast, T5_bin2, T3_bin6, and F8_bin2 show predominantly negative SHAP values at high feature values (red points), suggesting that increased values in these electrode–time bins reduce the model output. In contrast, lower values (blue points) are associated with positive contributions. Similar negative patterns are also observed for features such as C3_bin1, C4_bin4, O2_bin5, Fp2_bin2, F8_bin5, and T3_bin0, indicating that the learned decision function depends on both increases and decreases in localized spatiotemporal activity.

Importantly, the electrodes identified as influential by SHAP, particularly Fp2, F8, T5/T3/T4, and O2, are highly consistent with the optimal electrode subsets reported in [37], where multi-objective evolutionary algorithms and greedy selection methods were used to identify diagnostically informative EEG channels for MCI detection. That study consistently highlighted frontal, temporal, and occipital channels, including Fp1/Fp2, F8, T5/T6, and O1/O2, as informative for MCI detection. The convergence between SHAP-derived spatial attributions and independently optimized electrode subsets provides strong support for the neurophysiological validity of the model’s learned representations. Frequency-specific interpretations are not warranted here because the bin identifier denotes an electrode–time-bin feature index within the segmented EEG window rather than a frequency-decomposed band. Nevertheless, the preserved spatiotemporal structure enables the identification of localized electrode–time patterns that contribute to the model output. In particular, the temporal electrodes T3, T4, and T5, which show prominent SHAP contributions, are anatomically located over the lateral temporal cortex and are functionally linked to memory-related processes. These regions lie in close proximity to the medial temporal lobe, which includes the hippocampus, one of the earliest structures affected during the progression of MCI and AD [38, 39]. In addition, the identification of prefrontal electrodes Fp2, F4, and F8, along with the occipital electrode O2, as key contributors in the SHAP analysis underscores the neurophysiological relevance of the model’s learned features, as these sites correspond to cortical regions implicated in cognitive functions that are frequently disrupted in MCI. The prefrontal cortex, which is involved in attentional control and executive function, and the occipital cortex, which supports visual integration, both show altered EEG patterns in individuals with MCI [40, 41]. Taken together, these findings indicate that the model focuses on biologically meaningful brain regions and support its potential utility for spatially informed and clinically reliable EEG-based early detection of MCI.

#### LIME explanations

**Fig. 9:**
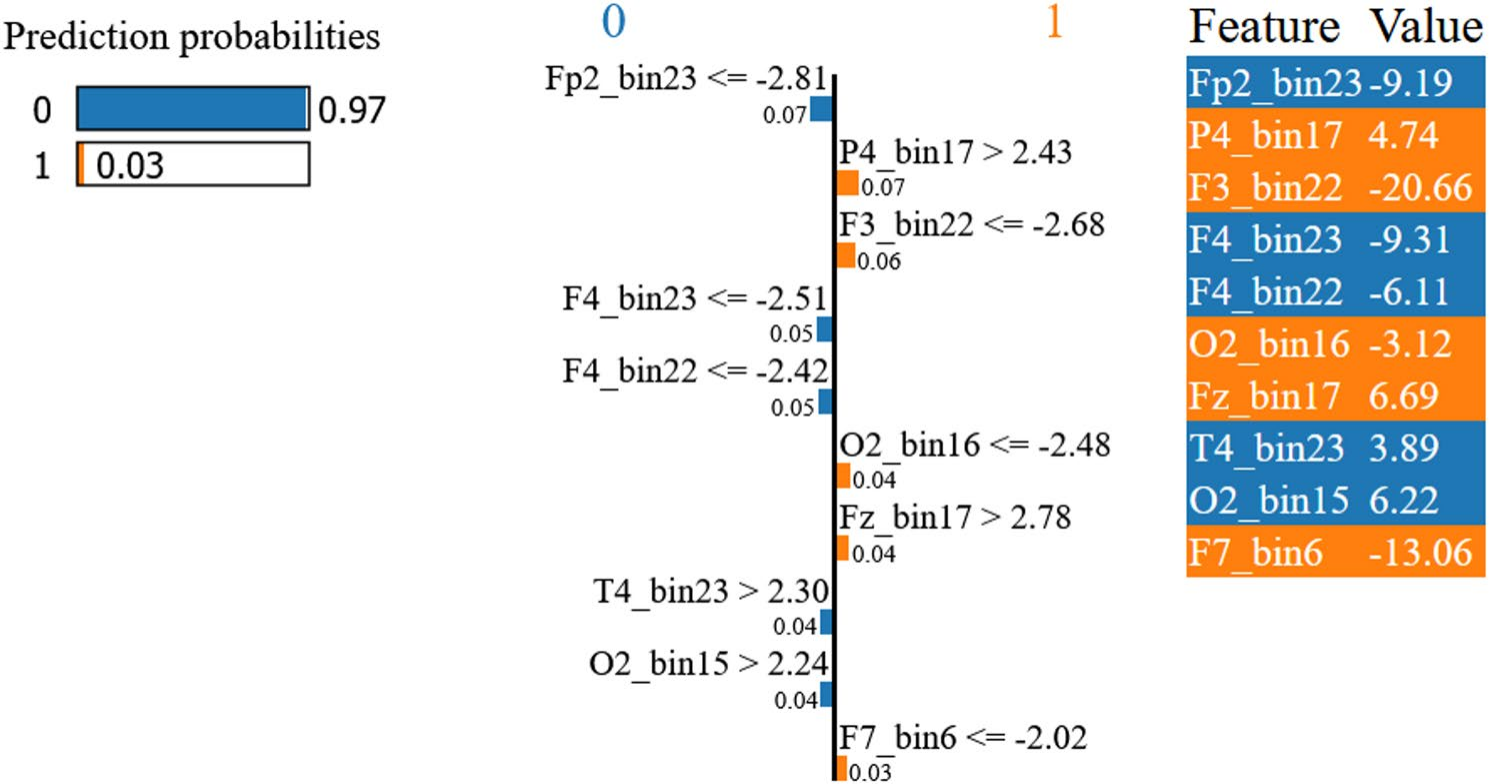
LIME explanation for HC segment 3233 from the primary resting-state dataset, showing the features that most strongly support or oppose the model’s prediction

To investigate the classifier’s decision-making process at the individual level, we applied LIME [42, 43] to two representative EEG segments. Segment 3233 (Fig. 9), classified as HC with a predicted probability of 0.97, was primarily supported by features contributing to class 0, most notably Fp2_bin23 ≤ − 2.81 (feature value: −9.19), F4_bin23 ≤ − 2.51 (− 9.31), and F4_bin22 ≤ − 2.42 (− 6.11). Additional evidence supporting the HC classification was provided by T4_bin23 > 2.30 (3.89) and O2_bin15 > 2.24 (6.22). In contrast, several features contributed counter-evidence toward class 1, including P4_bin17 > 2.43 (4.74), F3_bin22 ≤ − 2.68 (− 20.66), O2_bin16 ≤ − 2.48 (− 3.12), Fz_bin17 > 2.78 (6.69), and F7_bin6 ≤ − 2.02 (− 13.06). However, these contributions were not sufficient to outweigh the stronger evidence supporting the HC decision.

**Fig. 10:**
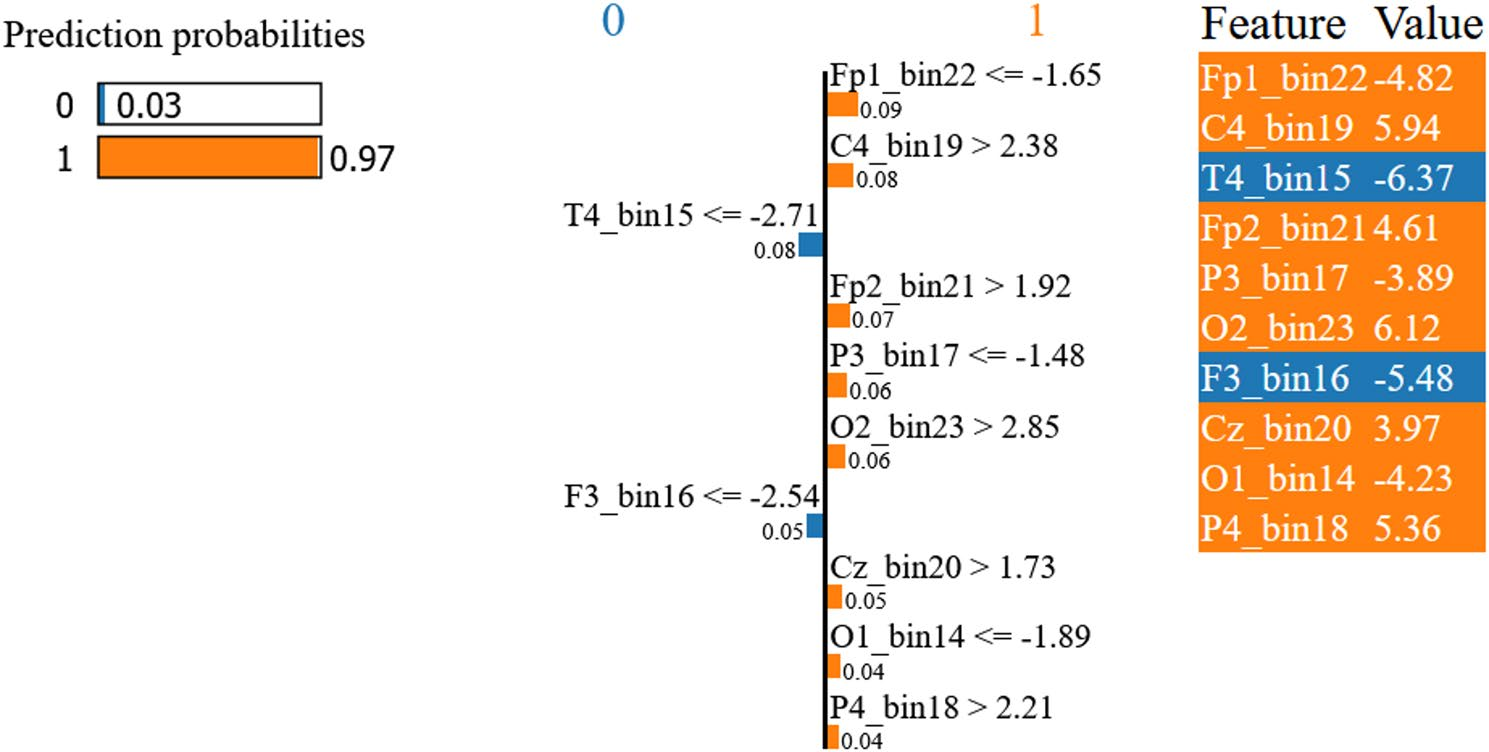
LIME explanation for MCI segment 5936 from the primary resting-state dataset, showing the features that most strongly support or oppose the model’s prediction

Sample 5936 (Fig. 10), classified as MCI with a predicted probability of 0.97, was supported by several features contributing to class 1, including Fp1_bin22 ≤ − 1.65 (− 4.82), C4_bin19 > 2.38 (5.94), Fp2_bin21 > 1.92 (4.61), P3_bin17 ≤ − 1.48 (− 3.89), O2_bin23 > 2.85 (6.12), Cz_bin20 > 1.73 (3.97), O1_bin14 ≤ − 1.89 (− 4.23), and P4_bin18 > 2.21 (5.36). At the same time, a smaller number of features provided counter-evidence toward class 0, most notably T4_bin15 ≤ − 2.71 (− 6.37) and F3_bin16 ≤ − 2.54 (− 5.48). This pattern indicates that the final MCI decision arises from the balance between supportive and opposing contributions from individual electrode–time-bin features. Because the feature index bin denotes a temporal position within the segmented EEG window rather than a frequency band, these explanations should be interpreted as spatiotemporal electrode–time effects rather than frequency-specific mechanisms.

Whereas the global SHAP analysis identified a stable set of spatially informative electrodes across the cohort, with prominent contributions from prefrontal and frontal sites (Fp2, F4, F7, F8), temporal sites (T3, T4, T5), and posterior regions including occipital (O2) and parietal channels (P3, P4), LIME provides sample-specific insight into how individual electrode–time-bin features support or oppose particular predictions. For example, O2-related features act in opposite directions across the two cases: O2_bin15 supports the HC decision in Sample 3233, whereas O2_bin23 contributes to the MCI decision in Sample 5936. This illustrates that both the direction and magnitude of feature effects can vary across time bins and across individual instances. Taken together, SHAP provides a cohort-level view of globally influential features, whereas LIME reveals context-dependent decision factors for representative individual samples, thereby supporting the qualitative interpretability of the model’s behavior.

## Discussion

This study investigates automated MCI detection from EEG with particular emphasis on how evaluation protocol affects reported performance in small-cohort settings. The proposed CNN–Res–SE–BiLSTM–BiGRU framework integrates convolutional and residual feature extraction, SE-based channel recalibration, and complementary bidirectional recurrent modeling to capture spatiotemporal patterns associated with MCI. In addition to architectural design, the study emphasizes clinically relevant decision-making by calibrating predicted probabilities using temperature scaling and selecting operating thresholds using Youden’s J statistic on validation predictions, while also reporting fixed thresholds (0.35 and 0.50) to explicitly illustrate sensitivity–specificity trade-offs. Calibration and threshold selection are performed on validation folds only and then applied unchanged to the held-out test fold, reducing the risk of optimistic bias from test-set tuning.

### Comparison with prior work on the same resting-state dataset

A key observation from Table 8 is that all studies conducted on the same primary resting-state EEG dataset evaluate performance using segment-based cross-validation, where EEG segments (rather than subjects) are distributed across folds. Because multiple segments from the same subject may appear in both training and test folds, segment-wise evaluation can inadvertently exploit subject-specific characteristics and thus overestimate generalization to unseen individuals. Nevertheless, segment-based benchmarking remains valuable for fair model-to-model comparison when the protocol is consistent across studies. Under this same segment-wise five-fold protocol, the proposed model achieves very high discrimination (accuracy 0.974 ± 0.022; ROC-AUC 0.999 ± 0.001; PR-AUC 1.000 ± 0.000 with Youden thresholding), demonstrating performance that is comparable to the strongest methods reported in Table 8 on the identical dataset and evaluation scheme. These results confirm that the proposed hybrid design provides competitive segment-level separability relative to existing deep learning approaches on this benchmark.

### Subject-independent evaluation on the resting-state dataset

To assess clinical generalizability more directly, the same resting-state dataset was also evaluated under strict subject-wise (stratified group) five-fold cross-validation, ensuring that no subject contributes data to both training and test folds. Under this protocol, performance decreases substantially and becomes highly variable (accuracy 0.560 ± 0.161; ROC-AUC 0.574 ± 0.310; PR-AUC 0.546 ± 0.280 with validation-selected Youden thresholding). The pronounced fold-to-fold variance reflects inter-subject heterogeneity and limited subject counts per fold, and it underscores that strong segment-wise results do not necessarily translate to reliable subject-independent performance in small resting-state cohorts. This contrast also reinforces the broader implication of Table 7: evaluation design can materially influence conclusions about model performance and generalization.

### Odor-evoked dataset: subject-independent validation and comparison with prior study

To provide complementary subject-independent validation under a more controlled paradigm, the proposed model was evaluated on an odor-evoked EEG dataset using subject-independent five-fold cross-validation. In contrast to the resting-state benchmark—where strict subject-wise evaluation produced substantially weaker and more variable performance—the odor-evoked setting yielded strong and stable results (accuracy 0.956 ± 0.051, balanced accuracy 0.954 ± 0.043, ROC-AUC 0.971 ± 0.051, PR-AUC 0.934 ± 0.132 at the validation-selected Youden operating point). This contrast is clinically plausible: stimulus-locked sensory paradigms can provide more specific and sensitive signatures of early cognitive impairment than unconstrained resting-state activity, and olfactory dysfunction has been widely reported as an early marker in MCI/AD, making odor-evoked responses a particularly informative probe [44]. Moreover, the controlled, time-locked structure of odor-evoked recordings can reduce nuisance variability relative to resting-state EEG, supporting more reliable cross-subject generalization even with a compact four-channel montage. Consistent with Table 7, only one prior study [44] is available on this odor dataset; for direct comparability we report 5-fold results as well, but under the stricter subject-independent split, and achieve performance that is at par with the previously reported 5-fold setting.

### Threshold-independent discrimination, operating-point selection, and calibration

The experiments highlight the importance of separating threshold-independent measures of discrimination from threshold-dependent operating performance. Metrics such as ROC-AUC and PR-AUC capture ranking-based separability and therefore remain unchanged across thresholding strategies, whereas metrics such as accuracy, sensitivity, and specificity vary with the chosen operating point. Reporting Youden-selected thresholds alongside fixed thresholds (0.35 and 0.50) provides a practical mechanism to tune sensitivity-specificity trade-offs without retraining, while maintaining transparency about how decision rules affect clinical operating characteristics. Calibration behavior differed by protocol. Temperature scaling required substantially stronger adjustment under strict subject-wise evaluation on the resting-state dataset (T = 4.222 ± 1.143), consistent with overconfident probabilities under harder generalization conditions. In contrast, calibration on the odor dataset was milder (T = 0.561 ± 0.385), indicating more stable confidence estimates under subject-independent evaluation in the odor-evoked setting.

### Contributions of architectural components: baselines and ablations

Baseline comparisons and ablation studies clarify which design elements contribute most strongly to performance, particularly under subject-independent evaluation on the odor dataset. Hybrid CNN–recurrent baselines consistently outperform single-stream models, and the strongest hybrid baseline (CNN + BiLSTM + GRU-head) approaches but remains below the proposed model (accuracy 0.934 ± 0.074 vs. 0.956 ± 0.051). Ablation results further show that removing SE blocks or residual connections reduces operating performance, supporting the role of channel recalibration and residual learning in robust feature extraction. The largest degradation is observed when removing the BiGRU branch, which substantially reduces ROC-AUC and PR-AUC, indicating that this component contributes to intrinsic separability rather than only shifting the operating point.

### Interpretability analysis and scope of conclusions

Interpretability analyses were conducted on the primary dataset under the segment-wise setting, where the full multichannel montage supports electrode-level attribution and model discrimination is sufficiently stable for post-hoc explanation. UMAP projections of the learned latent embeddings indicate partial class separation with overlap (silhouette coefficient 0.6452), consistent with the subtle and heterogeneous nature of prodromal MCI. SHAP analysis identifies a consistent set of influential electrodes spanning frontal/prefrontal, temporal, and posterior regions, aligning with neurophysiologically plausible networks implicated in cognitive impairment, while LIME explanations for representative cases show that individual predictions arise from a balance of supportive and opposing electrode–time-bin contributions. Collectively, these analyses provide qualitative insight into segment-level model behavior and feature utilization; however, since they are generated under a subject-dependent protocol, they should be interpreted as explanatory patterns for segment-level decisions rather than definitive subject-independent biomarkers.

### Limitations and future directions

Several limitations follow from the experimental results and should be considered when interpreting the scope of the conclusions. First, the primary resting-state dataset contains a limited number of subjects, and strict subject-wise evaluation is therefore highly variable. Larger cohorts, ideally multi-center, are required to quantify subject-independent generalization reliably and to stabilize calibration and operating-point estimation. Second, the odor-evoked dataset uses only four channels and includes a relatively small number of MCI subjects, which limits fine-grained spatial inference and constrains the generality of conclusions across montages and acquisition protocols; nevertheless, the strong subject-independent performance suggests that stimulus-locked responses can remain informative even under reduced electrode setups. Third, attention in the present architecture is primarily applied in the spatial-channel dimension, without explicitly modeling the temporal-frequency structure. Recent works [45–47] on time-frequency attention suggest that emphasizing spectral and time-frequency characteristics can improve EEG feature representation for neurological disorder classification; accordingly, future work will investigate cross-domain attention strategies (e.g., joint channel–temporal–frequency attention) to better capture complementary spatial, temporal, and spectral biomarkers and potentially enhance MCI detection performance. In parallel, future research should expand calibration analyses and incorporate modeling strategies that explicitly account for inter-subject variability, further improving robustness under realistic clinical deployment. Moreover, while the current interpretability analysis provides electrode-level insights using established methods (UMAP/SHAP/LIME), future work should advance toward more clinically meaningful region/topography-based explanations by mapping attributions to functional brain regions and network-level descriptions, as advocated in recent studies [48, 49]. Overall, the results support strong segment-level benchmarking performance and robust subject-independent detection in the odor-evoked setting; however, conclusions about resting-state subject-independent generalization remain preliminary due to the small cohort and pronounced inter-subject heterogeneity.

**Table 8:** Comparison of MCI classification performance between the proposed model and existing methods using the same datasets

Study	Year	Method used	Training method	Accuracy (%)
Resting-state EEG dataset				
Kashefpoor et al. [7]	2016	Spectral features + Neurofuzzy + KNN	Segment-wise, 10-fold CV	88.89
Yin et al. [11]	2019	SWT + spectral-temporal features + SVM	Segment-wise, random split (60/20/20)	96.94
Alvi et al. [16]	2023	LSTM	Segment-wise, 5-fold CV	96.41
Alvi et al. [17]	2022	SWT + GRU	Segment-wise, 5-fold CV	95.51
Zhou et al. [20]	2024	STCGRU (CNN + BiGRU)	Segment-wise, 5-fold CV	98.1
**This study**	**-**	**CNN-Res-SE-BiLSTM-BiGRU + Temp. scaling + Youden’s J (with fixed-threshold comparison)**	**Segment-wise, stratified 5-fold CV**	**97.40 ± 2.20 (Youden)/99.90 ± 0.10 (0.35)/99.90 ± 0.10 (0.50)**
Odor-evoked EEG dataset				
Riaz et al. [44]	2025	Multibranch attention-based temporal-spectral CNN	Subject-wise, 5-fold CV	96.51
**This study**	**-**	**CNN-Res-SE-BiLSTM-BiGRU + Temp. scaling + Youden’s J (with fixed-threshold comparison)**	**Subject-independent, stratified 5-fold CV**	**95.60 ± 3.70 (Youden)/94.20 ± 3.40 (0.35)/91.80 ± 4.10 (0.50)**

## Conclusions

This work proposed a CNN-Res-SE-BiLSTM-BiGRU framework for EEG-based MCI detection, combining residual feature learning, SE-based channel recalibration, and complementary bidirectional temporal modeling. The approach incorporates temperature scaling for probability calibration and validation-based threshold selection (Youden’s J), enabling practical operating-point control without retraining. Across experiments, the model achieved competitive segment-level performance on the resting-state dataset and strong, stable subject-independent performance on the odor-evoked dataset under subject-independent 5-fold cross-validation (accuracy 0.956 ± 0.051, ROC-AUC 0.971 ± 0.051, PR-AUC 0.934 ± 0.132). The pooled confusion matrix (TN = 1411, FP = 86, FN = 28, TP = 804) indicates low false negatives with controlled false positives. Overall, the results support the feasibility of an EEG-based MCI screening model that combines high discrimination with calibrated decision-making. Future work will focus on validation in larger multi-centre cohorts and on extending attention beyond channel-wise recalibration to explicitly capture temporal–frequency structure through cross-domain attention mechanisms.

## Data Availability

All data utilized in this study were obtained from publicly accessible EEG datasets. The primary dataset is a resting-state EEG dataset collected at the cardiac catheterization units of Sina and Nour Hospitals (Isfahan, Iran) and contains recordings from 27 subjects (16 HC, 11 MCI). In addition, we used an independent odor-evoked EEG dataset (Sedghizadeh et al., Data Brief 2023) collected from 35 subjects consisting of 13 AD, 7 aMCI (amnestic MCI), and 15 HC participants.
